# Iron repletion is associated with reduction in platelet counts in non-dialysis chronic kidney disease patients independent of erythropoiesis-stimulating agent use: a retrospective cohort study

**DOI:** 10.1186/1471-2369-15-119

**Published:** 2014-07-19

**Authors:** Lenar Yessayan, Jerry Yee, Gary Zasuwa, Stan Frinak, Anatole Besarab

**Affiliations:** 1 Division of Nephrology and Hypertension, Henry Ford Hospital, 2799 W. Grand Blvd, CFP-514, Detroit, MI, USA; 2 Division of Nephrology and Hypertension, University of California, San Francisco, CA, USA

**Keywords:** Thrombocytosis, Platelet count, Iron deficiency anemia, Erythropoietin, Intravenous iron dextran

## Abstract

**Background:**

Iron deficiency is common in non-dialysis chronic kidney disease (ND-CKD) patients and, on occasion, requires parenteral iron therapy. We investigated the effect of intravenous iron repletion on platelet counts in ND-CKD patients with and without concomitant darbepoetin administration.

**Methods:**

We conducted a retrospective analysis of ND-CKD patients with iron deficiency anemia treated with low molecular weight iron dextran (LMWID) between 2005 and 2009 at our CKD clinic. The primary end-point was change in platelet count 60 days post infusion of LMWID in those with and without concomitant darbepoetin administration. Secondary end-points were the correlations between changes in platelet count and iron indices.

**Results:**

A total of 108 patients met inclusion and exclusion criteria. The decrease in platelet counts in response to iron repletion was statistically significant (305.72 ± 108.86 vs 255.58 ± 78.97, P = < .0001). The decrease in platelet count was independent of concomitant darbepoetin use. Bivariate regression analysis between baseline platelet count and transferrin saturation by iron (TSAT) showed a negative association (β_TSAT_ = −5.82, P = .0007) and moderate correlation (*R* = 0.32). Following iron treatment, the within individual changes in platelet count in 60 days were not related to changes in TSAT (β_ΔTSAT_ = −0.41, P = .399) and demonstrated a poor correlation (*R* = 0.10).

**Conclusions:**

Parenteral iron treatment by LMWID is associated with reduction in platelet counts in iron deficient anemic ND-CKD patients. However, ESA use in the majority of patients prior to intravenous iron administration could have altered platelet production through bone marrow competition.

## Background

Thrombocytosis may be the result of a clonal disorder or secondary to a reactive process such as acute or chronic inflammation, hyposplenism, malignancy, or iron deficiency. The mechanism of thrombocytosis in iron deficiency related anemia is not completely understood. Thrombocytopoiesis is orchestrated by a complex interplay between growth factors and cytokines. Thrombopoietin is the primary growth factor and regulator of megakaryopoiesis [1]; however, several other cytokines such as interleukin (IL)-1, IL-3, IL-6, IL-11 and tumor necrosis factor are involved in this process [2-7]. Erythropoietin shares some structural features with thrombopoietin and may exhibit synergistic effect on platelet production [8,9]. However, the small degree of functional overlap is not mediated by cross-reactivity at the level of mpl receptor expressed on the surface of megakaryocytes [10]. Treatment with exogenous erythropoietin may increase platelet count [11]. It may be that the mechanism of synergy is mediated through the expression of erythropoietin receptors on megakaryocyte progenitors. It has also been suggested that the mechanism by which erythropoiesis stimulating agents (ESA) increase platelet count may be through the induction of a state of functional iron deficiency [12]. Some have suggested that reactive thrombocytosis, induced by ESA or the resulting iron depletion, may have contributed to the higher-than-expected rates of cerebrovascular accidents and thrombotic events reported in clinical trials of ESA in chronic kidney disease (CKD) patients. At the Henry Ford Hospital Chronic Kidney Disease Clinic, low molecular weight iron dextran (LMWID) in conjunction with ESA is used for non-dialysis chronic kidney disease (ND-CKD) anemia management using a computerized algorithm for dosing. We undertook the present study to evaluate the temporal effect of intravenous iron administration on platelet counts in iron deficient ND-CKD patients and determine if the effect is dependent on concurrent ESA administration.

## Methods

This single-center retrospective cohort study was approved by our hospital’s institutional review board (IRB project number 6650; Henry Ford Health Systems Institutional Review Board). We performed a chart review of all ND-CKD patients enrolled in our CKD clinic’s computerized anemia management program (CAMP) [13] who received iron dextran (INFeD, Watson Pharmaceuticals, Parsippany, NJ) between August 25, 2005 and March 6, 2009. CAMP is designed to treat anemia of CKD using darbepoetin alpha (DA) and iron treatment algorithms. Data on patients’ hematologic variables are stored in the CAMP database irrespective of whether they received the recommended dose of iron/ESAs. After manual data input, the iron dosing algorithm recommends no iron, oral iron, or 1 g of LMWID over 1.5–2 hours based on hemoglobin, TSAT, and ferritin levels obtained within 30 days of iron infusion. At all times, iron “sufficiency” is to be achieved/maintained with oral or parenteral ID per the algorithm [14]. The dosing of LMWID (500 or 1000 mg) during this study period was at the discretion of the treating physician. DA doses were determined monthly, based on three separate protocols for first, second, and maintenance DA doses with the goal to achieve and maintain Hb in the range of 10.0–12.0 g/dL [13].

Eligible patients were adults (≥18 years) with CKD who received a single dose of LMWID during a 2-month period and who were not on maintenance ESA therapy prior to LMWID administration. Exclusion criteria were any of the following: documented surgery, gastrointestinal bleeding, hospitalization during the evaluation period; lack of follow-up on second evaluation (within 37 days from baseline) or on third evaluation (within 37 days from second evaluation); administration of ESAs within 30 days of LMWID infusion. Of the 131 subjects identified, 108 ND-CKD patients met eligibility criteria. All of these study subjects received either a 500 mg or 1000 mg infusion by peripheral vein over 1.5–2.0 hours. No patient received a LMWID test dose and none were premedicated with diphenhydramine. We previously reported that LMWID is safe and effective in the treatment of iron deficiency in ND-CKD [14]. However, because fatal anaphylactic reactions have been reported after administration of LMWID injection, resuscitation techniques and treatment of anaphylactic and anaphylactoid shock are readily available in our CKD clinic.

Baseline platelet counts, hemoglobin and iron indices were obtained within 1 week prior to LMWID administration, at 30 and 60 days post LMWID administration. The primary end-point was the change in platelet count at ~ 60 days from iron administration (baseline) in those with and without concomitant DA administration. Secondary end-points were changes in iron indices at ~ 30 and 60 days from baseline and the correlation between changes in iron indices and platelet count following LMWID administration. To detect a reduction in platelets of 25 000/mm^3^ with a two-sided 5% significance level and a power of 80%, a sample size of at least 60 patients was necessary to account for an anticipated 20% exclusion, either because of lack of follow-up or presence of intercurrent events. To include at least this number of patients, a 48-month inclusion period was considered.

Statistical analysis was performed using SAS software version 9.3 (SAS Institute, Inc., Cary, NC). We used Student’s paired *t*-test to measure intra individual changes between time points for platelets, ferritin, and TSAT. Pearson correlation coefficients were used to evaluate the relationship between platelet count and iron indices at baseline as well as between changes in platelet counts and iron indices 60 days post-administration. Categorical variables are presented as frequencies and percentages. Continuous variables are presented using mean ± standard deviation. For all analyses, a P value < .05 was considered significant.

## Results

**Figure 1:**
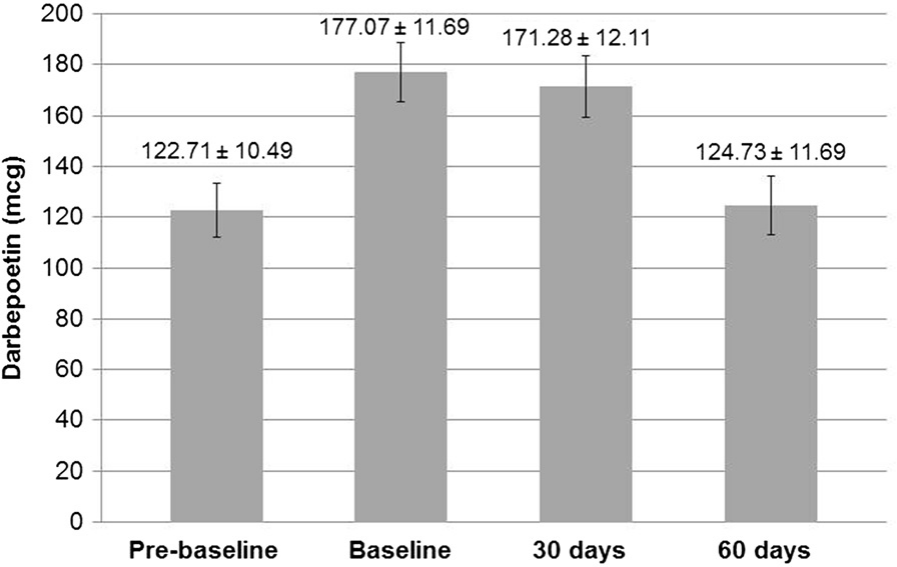
**Darbepoetin alfa (mcg) ± SD utilization over duration of study in the group that received Darbepoetin alfa.** Pre-baseline indicates within 30 days of iron infusion. Baseline indicates the day of iron infusion.

Table 1 shows demographics and baseline laboratory values of 108 patients receiving LMWID infusions stratified by concomitant DA use. All CKD stages were represented (Table 1). Stages 3 and 4 CKD were the most common and CKD stage 1 was the least common. There were 65 (60%) 1000 mg LMWID infusions and 43 (40%) 500 mg LMWID infusions administered. Of the 108 subjects, 94 received monthly DA injections either on the day of iron infusion or 30 days prior to iron infusion or both; and 14 patients did not receive any DA within 30 days prior to iron infusion or during the evaluation period. Figure 1 shows the mean monthly doses of DA over time in the DA group.

**Table 1:** Baseline characteristics of patients receiving intravenous iron dextran stratified by darbepoetin exposure

	**Darbepoetin**	**No darbepoetin**	**P value**
	**N = 94**	**N = 14**	
Demographics			
Age	69.51 ± 15.90	67.79 ± 17.26	0.7088 (T)
Race, *n* (White, Black, Asian, Hispanic)	45/47/1/1	13/1/0/0	0.0181 (F)
Gender, *n* (Female/Male)	58/36	11/3	0.2202 (C)
Laboratory			
Baseline platelets (1000/mm^3^)	304.53 ± 106.97	313.71 ± 124.87	0.7699 (T)
Hemoglobin (g/dL)	9.98 ± 1.22	11.44 ± 1.43	<0.0001 (T)
Transferrin saturation (%)	13.07 ± 6.26	12.71 ± 4.41	0.8361 (T)
Ferritin (ng/mL)	43.07 ± 36.05	33.50 ± 23.10	0.3379 (T)
CKD stage			
Stage 1	1 (1%)	0 (0%)	0.4453 (C)
Stage 2	6 (6%)	0 (0%)	
Stage 3	40 (43%)	9 (64%)	
Stage 4	38 (40%)	5 (36%)	
Stage 5	9 (10%)	0 (0%)	

Demographics are reported as frequencies except for age which is reported as mean ± standard deviation. Laboratory parameters are reported as mean ± standard deviation. Chronic kidney disease (CKD) stages are reported as frequencies with percentages in parenthesis. (T) = Two-sided T-Test, (C) = Chi-Square Test, (F) = Fisher Exact Test.

Following administration of LMWID, hemoglobin, ferritin, and iron saturation increased significantly at 30 and 60 days. Concomitantly, at 30 and 60 days, a statistically significant decrease occurred in platelet counts in response to iron repletion (Table 2). When platelet response was stratified according to DA use (Table 3), there was a statistically significant decrease in platelet counts from baseline at 30 days in both groups (DA group 304.53 ± 106.97 vs 274.26 ± 136.27, P = 0.008; No DA group 313.71 ± 124.87 vs 279.00 ± 130.08, P = 0.0021). At 60 days, a statistically significant decrease in platelet counts of approximately 50 000/mm^3^ was observed in both groups in response to iron repletion. The change in platelet count was similar when stratified according to DA use (−50.12 ± 63.36 for DA group vs −50.29 ± 74.64 for No DA group, P = .9928). Irrespective of iron dose, a decrease in platelet count was observed (Table 4). The change in platelet count 60 days post-LMWID was similar when platelet response was stratified according to dose of LMWID administered (−50.33 ± 61.40 for 500 mg vs −50.02 ± 67.02 for 1000 mg; P = .9806).

**Table 2:** Parameter comparison at baseline, 30 days and 60 days post-LMWID administration

**Parameter**	**Pre-infusion**	**30 days**	**p-value**	**60 days**	**p-value**
	**Mean ± SD**	**Mean ± SD**		**Mean ± SD**	
Platelet count (1000/mm^3^)	305.72 ± 108.86	274.71 ± 135.04	0.0012	255.58 ± 78.97	<0.0001
Hemoglobin (g/dL)	10.17 ± 1.34	11.29 ± 1.66	<0.0001	11.38 ± 1.70	<0.0001
Ferritin (ng/mL)	41.83 ± 34.71	203.29 ± 168.75	<0.0001	154.13 ± 156.39	<0.0001
Transferrin saturation (%)	13.03 ± 6.03	23.62 ± 12.60	<.00001	25.06 ± 13.30	<0.0001

**Table 3:** Parameter comparison pre-LMWID and post-LMWID infusion stratified by darbepoetin administration

**Darbepoetin (N = 94)**					
**Parameter**	**Pre-infusion**	**30 days**	**p-value**	**60 days**	**p-value**
	**Mean ± SD**	**Mean ± SD**		**Mean ± SD**	
Platelet count (1000/mm^3^)	304.53 ± 106.97	274.26 ± 136.27	0.008	254.41 ± 80.16	<0.0001
Hemoglobin (g/dL)	9.98 ± 1.22	11.12 ± 1.61	<0.0001	11.22 ± 1.70	<0.0001
Ferritin (ng/mL)	43.07 ± 36.05	208.06 ± 171.79	<0.0001	150.58 ± 151.79	<0.0001
Transferrin saturation (%)	13.07 ± 6.26	24.32 ± 12.83	<0.0001	25.83 ± 13.55	<0.0001
**No Darbepoetin (N = 14)**					
**Parameter**	**Pre-infusion**	**30 days**	**p-value**	**60 days**	**p-value**
	**Mean ± SD**	**Mean ± SD**		**Mean ± SD**	
Platelet count (1000/mm^3^)	313.71 ± 124.87	279.00 ± 130.08	0.0021	263.43 ± 72.68	0.0256
Hemoglobin (g/dL)	11.44 ± 1.43	12.66 ± 1.51	0.0049	12.46 ± 1.30	0.0053
Ferritin (ng/mL)	33.50 ± 23.10	135.00 ± 103.01	0.0276	192.25 ± 208.21	0.0620
Transferrin saturation (%)	12.71 ± 4.41	16.00 ± 6.07	0.1047	16.75 ± 5.92	0.0683

LMWID = low molecular weight iron dextran.

**Table 4:** Parameter comparison pre-LMWID and 60 days post-LMWID infusion stratified by iron dextran dose

	**Iron dextran 1000 mg**			**Iron dextran 500 mg**		
	**N = 65**			**N = 43**		
	**Pre-infusion**	**Post-infusion**	**P value**	**Pre-infusion**	**Post-infusion**	**P value**
	**Mean ± SD**	**Mean ± SD**		**Mean ± SD**	**Mean ± SD**	
Platelet count (1000/mm^3^)	323.54 ± 118.82	273.52 ± 87.34	<0.0001	278.79 ± 86.27	228.47 ± 54.87	<0.0001
Hemoglobin (g/dL)	10.24 ± 1.41	11.60 ± 1.63	<0.0001	10.07 ± 1.23	11.04 ± 1.78	0.0001
Ferritin (ng/mL)	46.22 ± 38.31	174.60 ± 158.25	<0.0001	35.21 ± 27.54	122.59 ± 150.15	0.0005
Transferrin saturation (%)	13.60 ± 6.43	24.95 ± 12.12	<0.0001	12.16 ± 5.34	25.24 ± 15.07	<0.0001

LMWID = low molecular weight iron dextran.

Bivariate regression analysis between the baseline platelet count and TSAT revealed a negative association between the 2 variables. The estimated baseline platelet count increased by ≈ 5820 for each 1% decrease in TSAT (β_TSAT_ = −5.82, P = .0007). The correlation coefficient *R* between baseline platelet and TSAT was 0.32. No association was found between baseline platelet count and ferritin (β_ferritin_ = −0.32, P = .2838), and the correlation coefficient *R* was not significant (*R* = 0.10, P = .2838).

**Figure 2:**
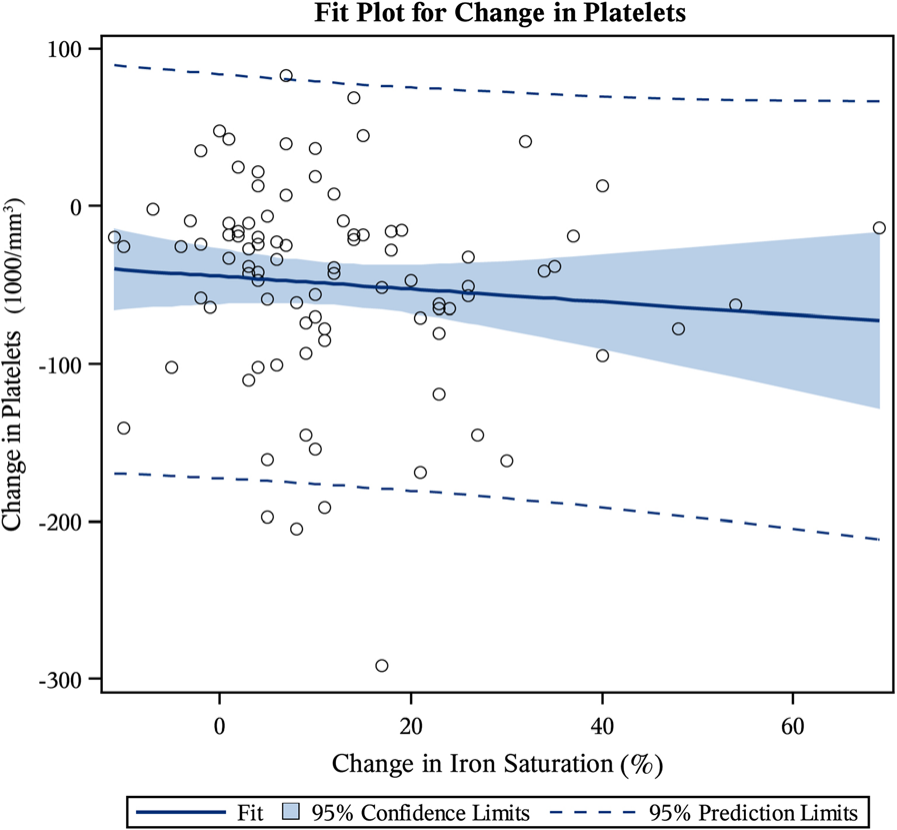
Regression fit plot of within individual changes in platelets versus changes in transferrin saturation.

**Figure 3:**
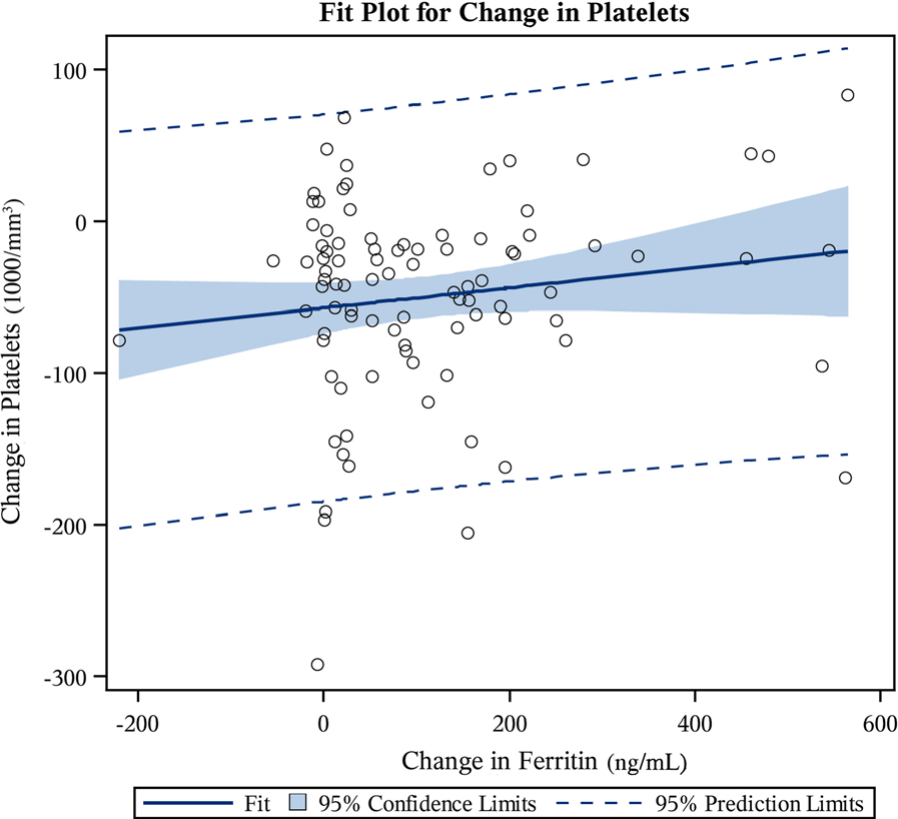
Regression fit plot of within individual changes in platelets versus changes in ferritin.

The linear correlation between changes in platelet counts and iron indices in response to LMWID at 60 days was also investigated. The change in platelet count in ~ 60 days was neither related to changes in iron saturation (β_ΔTSAT_ = −0.41, P = .3991) nor ferritin (β_ferritin_ = 0.07, P = .1458), as displayed in Figures 2 and 3, respectively. Both predictor variables exhibited a weak and statistically non-significant correlation with platelet change (*R* = −0.09, *R* = −0.15, respectively). To test the robustness of this result, we repeated this analysis after the exclusion of patients who did not have any improvement in their TSAT by 60 days from baseline. The poor correlation persisted even after the exclusion of these patients (*R* = −0.07934).

## Discussion

Secondary thrombocytosis is associated with many conditions, including acute or chronic inflammation, hyposplenism, and iron deficiency. Although generally regarded as a benign condition, secondary thrombocytosis has been identified as an independent risk factor for thromboembolic events in patients with cancer [15-17]. Similarly, iron deficiency with and without thrombocytosis has been linked to venous thrombosis and stroke [18-20]. Thus, it is biologically plausible that reactive thrombocytosis, induced by ESA or the resulting iron depletion, may have contributed to the higher-than-expected rates of cerebrovascular accidents and thrombotic events reported in clinical trials of ESA in CKD patients (e.g., the cardiovascular risk reduction by early anemia treatment with epoetin beta [CREATE] trial, a trial of end-stage renal disease patients that compared the effects of normal as compared with low hematocrit values in patients with cardiac disease who were receiving hemodialysis and epoetin, and a trial to reduce cardiovascular events with Aranesp therapy [TREAT]) [21-23] as postulated by Streja et al. [24]. In their retrospective analysis of more than 40 000 hemodialysis patients, Streja et al. [24] noted relative thrombocytosis in 15% (defined as platelet count > 300 000 ± 109 000/L), and this was associated with a 30% greater weekly dose of ESA, lower TSAT, and lower serum ferritin concentration. This pattern is reflective of functional iron deficiency at the bone marrow level.

Megakaryopoiesis is regulated by various cytokines [1-7]. The pathophysiologic mechanism behind reactive thrombocytosis in iron deficiency is complex and incompletely understood. Akan et al. [25] demonstrated that the correction of iron deficiency anemia and resolution of thrombocytosis do not alter cytokine levels that are typically elevated in reactive thrombocytosis (IL-6, IL-11, and thrombopoietin). Although the levels of endogenous erythropoietin significantly decreased during correction of iron deficiency, the same response was observed in those with and without thrombocytosis, indicating that erythropoietin is not the principal regulator of thrombocytosis [25].

Studies of intravenous iron undertaken in predialysis chronic renal failure that evaluated hematologic parameters have not reported the effect on platelet counts [26-30]. This observational study evaluated the effect of intravenous LMWID on platelet counts in iron deficient anemic ND-CKD patients with and without concomitant ESA use. We also evaluated the relationship between baseline platelets and iron storage parameters, as well as the relationship between changes in iron storage parameters and platelets. In response to iron administration, a statistically significant decrease occurred in platelet counts of about 50 000/mm^3^, suggesting a mechanistic link between iron deficiency and platelet counts in patients with ND-CKD. The effect of iron repletion on platelet count was observed in those who did and did not receive ESAs suggesting that the effect of iron repletion on platelet counts is independent of ESA use.

In our study the correlation between baseline platelet and TSAT was weak to moderate and negative in direction (*R* = −0.31); this is consistent with previously reported negative associations between baseline iron stores and platelet counts. One recent study showed only a very weak correlation between baseline platelet count and iron stores and no reduction in platelet count after intravenous iron dextran [31]. However, contrary to our study, the after dose values were measured between 10–120 days. Interestingly, when we investigated the relationship of within individual change in platelet count in response to a change in TSAT, we only found a weak and insignificant correlation. This would imply that our current parameters for iron deficiency, TSAT and ferritin, may be insensitive and imprecise measures of iron availability to the bone marrow. To date, the only definitive way in clinical practice to prove the presence of iron deficiency in CKD is to evaluate the erythropoietic response to parenteral iron administration. However, both reticulocyte hemoglobin content (CHr) and the percentage of hypochromic red cells may be better indicators of iron deficiency (CHr area under the curve [AUC] = 0.935 for a cutoff of 29.8 pg, sensitivity 90.7%, specificity 83.1%; percent hypochromic cells AUC = 0.925, cutoff 3.5%, sensitivity 87.3%, and specificity 88.0%) [32] than the traditional measurements of TSAT and ferritin, which have sensitivities and specificities < 80%.

This study has several limitations. Its retrospective nature makes it subject to selection and information bias. Selection bias was constrained by including all patients within the prespecified dates; however, some patients were excluded because of a lack of follow-up or intercurrent events. Moreover, given the retrospective design, patients with undocumented intercurrent events in our records such as gastrointestinal bleed, infection and surgery may have been included in the study and caused bias in the point estimate of the correlation. However, an uncertainty analysis that was performed with the exclusion of those who did not have any improvement in their TSAT yielded similar results. Additionally, by design, we could not fully control information bias; however, any observer bias was limited by data abstraction from the anemia management database. This study suggests that iron replacement is associated with reduction in platelet counts in iron deficient ND-CKD patients independent of ESA use and extends the observation of Eschbach’s study [33], where it was noted that almost half of their 333 study patients developed iron deficiency with platelet counts increasing following erythropoietin administration to anemic end-stage renal disease patients. Aggressive iron therapy reversed this increase in platelets.

Another limitation was the absence of data regarding inflammatory markers, such as C-reactive protein and erythrocyte sedimentation rate, and for this reason, we could not adjust for the effect of inflammatory status, if any, on platelet count change. Furthermore, the majority of our patients did not have thrombocytosis (median = 284 000, interquartile range = 236–359). The decrease in platelet count following LMWID could only be extrapolated to patients within the specified platelet count range in our cohort. Whether iron administration could induce a more pronounced change in platelet count in iron deficient patients with thrombocytosis warrants further investigation.

## Conclusion

In conclusion, our study implies that intravenous iron is associated with reduction in platelet counts independent of ESA use. However, without a control group not receiving IV iron, causation cannot be confirmed. Adequately powered studies are needed to establish an association between thrombocytosis, iron deficiency and thromboembolic events in ND CKD patients.

## Abbreviations

AUC: Area under the curve; CAMP: Computerized anemia management program; CHr: Reticulocyte hemoglobin content; DA: Darbepoetin; ESA: Erythropoietin stimulating agent; IL: Interleukin; LMWID: Low molecular weight iron dextran; ND-CKD: Non-dialysis chronic kidney disease; TSAT: Transferrin saturation.

## Competing interests

L. Yessayan has nothing to disclose. J. Yee, A. Besarab, S. Frinak and G. Zasuwa are inventors of Computerized Algorithm Management Program (CAMP). A. Besarab has received research funds, grants, or contracts from Affymax, Amgen Inc., Hoffman-La Roche Ltd., Rockwell International, Takeda Pharmaceuticals, VascAlert, Fibrogen Inc., Watson Pharmaceuticals, and Bayer Pharma. J. Yee is a consultant for Amgen, Affymax, Merck, and Alexions and a shareholder in Merck.

## Authors’ contributions

AB is the principal investigator and conceived the study. LY, JY, GZ and SF contributed to study design. JY, GZ and SF acquired the data. LY, JY and AB analyzed and interpreted the data. LY, JY and AB wrote the manuscript. LY, JY, and AB revised the manuscript for important intellectual content. All authors read and approved the final manuscript.

## Pre-publication history

The pre-publication history for this paper can be accessed here:

http://www.biomedcentral.com/1471-2369/15/119/prepub
